# Rubella virus assembly requirements and evolutionary relationships with novel rubiviruses

**DOI:** 10.1128/mbio.01965-24

**Published:** 2024-08-29

**Authors:** Pratyush Kumar Das, Margaret Kielian

**Affiliations:** 1 Department of Cell Biology, Albert Einstein College of Medicine, Bronx, New York, USA; Columbia University, New York, New York, USA

**Keywords:** rubella virus, rubivirus, virus assembly, virus budding

## Abstract

Rubella virus (RuV) is an enveloped virus that usually causes mild disease in children, but can produce miscarriage or severe congenital birth defects. While in nature RuV only infects humans, the discovery of the related Ruhugu (RuhV) and Rustrela (RusV) viruses highlights the spillover potential of mammalian rubiviruses to humans. RuV buds into the Golgi, but its assembly and exit are not well understood. We identified a potential late domain motif ^278^PPAY^281^ at the C-terminus of the RuV E2 envelope protein. Such late domain motifs can promote virus budding by recruiting the cellular ESCRT machinery. An E2 Y281A mutation reduced infectious virus production by >3 logs and inhibited virus particle production. However, RuV was insensitive to inhibition by dominant-negative VPS4, and thus appeared ESCRT-independent. The E2 Y281A mutation did not significantly inhibit the production of the viral structural proteins capsid (Cp), E2, and E1, or dimerization, glycosylation, Golgi transport, and colocalization of E2 and E1. However, E2 Y281A significantly reduced glycoprotein-Cp colocalization and interaction, and inhibited Cp localization to the Golgi. Revertants of the E2 Y281A mutant contained an E2 281V substitution or the second site mutations [E2 N277I + Cp D215A]. These mutations promoted virus growth, particle production, E2/Cp colocalization and Cp-Golgi localization. Both the E2 substitutions 281V and 277I were found at the corresponding positions in the RuhV E2 protein. Taken together, our data identify a key interaction of the RuV E2 endodomain with the Cp during RuV biogenesis, and support the close evolutionary relationship between human and animal rubiviruses.

**IMPORTANCE:**

Rubella virus (RuV) is an enveloped virus that only infects humans, where transplacental infection can cause miscarriage or congenital birth defects. We identified a potential late domain, ^278^PPAY^281^, at the C terminus of the E2 envelope protein. However, rather than this domain recruiting the cellular ESCRT machinery as predicted, our data indicate that E2 Y281 promotes a critical interaction of the E2 endodomain with the capsid protein, leading to capsid's localization to the Golgi where virus budding occurs. Revertant analysis demonstrated that two substitutions on the E2 protein could partially rescue virus growth and Cp-Golgi localization. Both residues were found at the corresponding positions in Ruhugu virus E2, supporting the close evolutionary relationship between RuV and Ruhugu virus, a recently discovered rubivirus from bats.

## INTRODUCTION

Rubella virus (RuV) is a positive-sense single-stranded RNA virus that belongs to the *Rubivirus* genus in the *Matonaviridae* family (1). RuV generally causes mild symptoms in children and adults, but infection in pregnant women can lead to transplacental fetal infection, resulting in miscarriage or a variety of severe birth defects known collectively as congenital rubella syndrome (CRS) (2, 3). Humans are the only known host for RuV in nature, and although RuV is highly contagious through aerosol transmission, efficacious vaccines have eliminated the endemic infection in many parts of the world including the Americas (4, 5). Nonetheless, worldwide, around 100,000 cases of CRS are still reported each year (6). For many years, RuV was the only known member of the *Rubivirus* genus. Recently, metagenomic analyses identified Ruhugu virus (RuhV) and Rustrela virus (RusV) as two novel mammalian rubiviruses (7). RuhV was identified in apparently healthy bats in Uganda; potential spillover hosts are unknown (7). RusV was found in several species of apparently healthy mice in northern Europe, and was identified in a variety of animals suffering from lethal encephalitis and neurological disease, including capybaras, donkeys, wallabies, coatis, otters, and cats (7–9). The viruses themselves have not yet been isolated. The discovery of these novel mammalian rubiviruses raises concerns about their potential spillover to humans.

**Fig 1:**
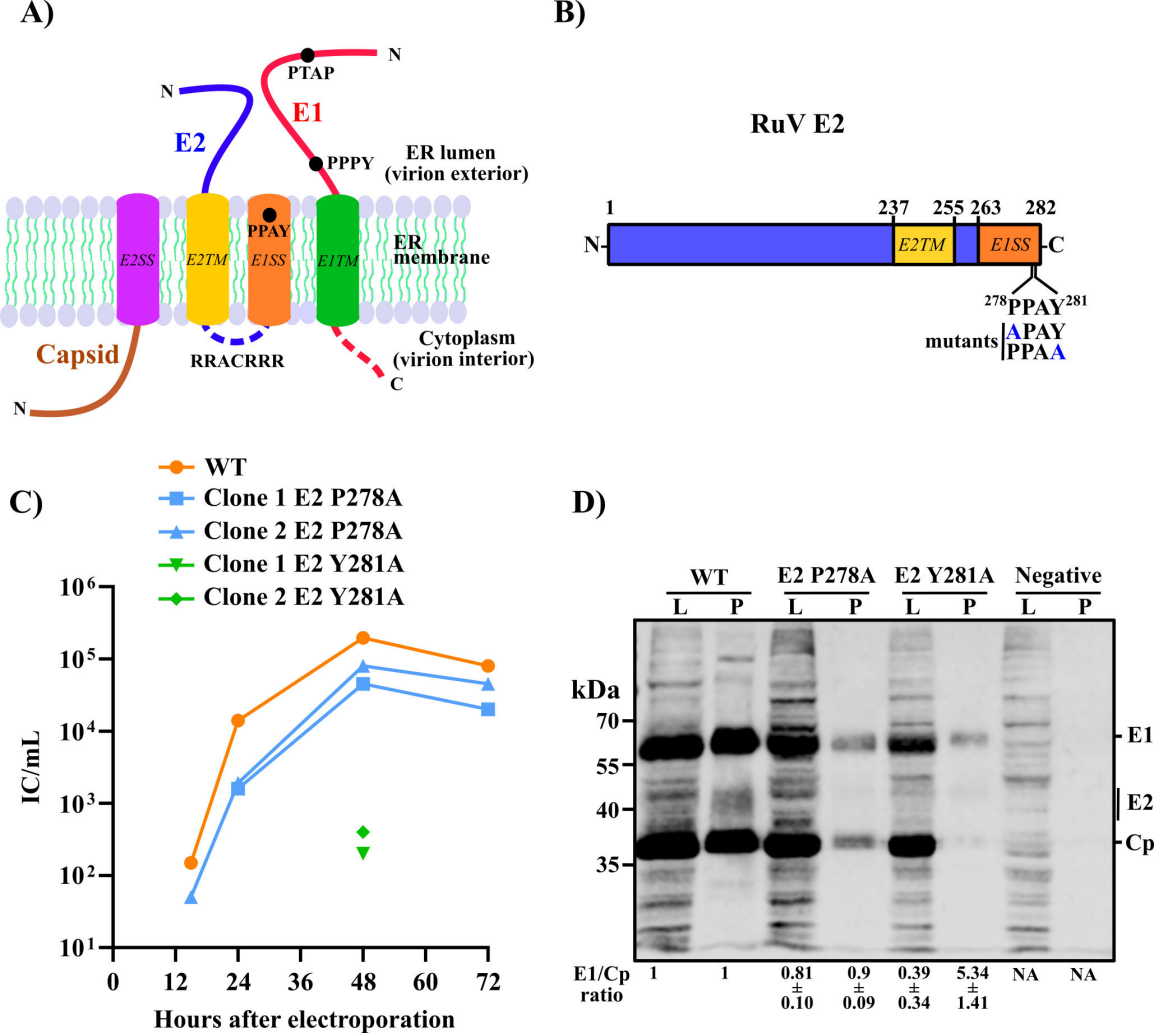
Identification and mutagenesis of a potential RuV E2 late domain motif. (**A**) Putative model for the topological arrangement of the RuV structural proteins on the ER membrane, showing potential late domain motifs. Capsid (umber) remains attached to the ER membrane by the E2SS (magenta); glycoprotein E2 (blue) and E1 (red) are membrane-anchored by the indicated TM domains on E2 (yellow) and E1 (green). The E2TM and E1SS (shown in orange) are connected by an arginine-rich cytoplasmic region (dotted blue line). The E1 cytoplasmic domain is shown as a dotted red line. Potential late domain motifs on the E1 ectodomain (PTAP and PPPY) and the E2 C-terminal region (PPAY) were identified bioinformatically and their positions are indicated by circles. (**B**) Schematic showing E2 domains colored as in (**A**) with residue numbers indicated. The PPAY motif is present at the E2 C-terminus followed by the last E2 aa. The E2 P278A and E2 Y281A alanine substitution mutants designed for this study are indicated in blue. (**C**) Growth kinetics of WT RuV and E2 mutants. BHK-21 cells were electroporated with *in vitro* transcribed WT or mutant RNAs from two independent clones. Culture media were harvested at the indicated time points and virus was quantitated by infectious center assay (ICA). Infectious E2 Y281A virus was only detected at the 48 h time point. (**D**) Particle production of WT and E2 mutants. BHK-21 cells were electroporated with WT or mutant RNA, cultured for 48 h, and lysates prepared. The clarified culture media were pelleted through a sucrose cushion. Samples were analyzed by SDS-PAGE and western blotting with RuV pAb. L: lysate, P: pelleted medium. The mean E1/Cp ratio ±range (*n* = 2) is indicated under each lane relative to WT, set as 1. Note the E2 heterogeneity due to post-translational modifications (20), and its relative inefficient recognition by the RuV pAb.

RuV is a pleomorphic enveloped virus with a 10 kb RNA genome encoding the p200 nonstructural polyprotein that mediates viral RNA replication, and the p110 structural polyprotein (2, 10–12). p110 is processed by signal peptidase in the endoplasmic reticulum (ER) to produce the capsid protein (Cp) and the E2 and E1 envelope proteins (see Fig. 1A) (2, 11). The signal sequence (SS) for E2 translocation into the ER remains attached to the C-terminus of Cp and confers Cp-membrane binding (13, 14). The E2 and E1 envelope glycoproteins are anchored by transmembrane (TM) domains. It is believed that, similar to the E2SS serving as a TM anchor for the Cp, the E1SS at the C-terminus of E2 also remains integrated in the membrane, but direct evidence to support this is lacking (2, 11, 15). The Cp interacts with the viral genomic RNA to form the nucleocapsid (NC) core, which is enveloped by a lipid bilayer containing E2-E1 heterodimers (2, 10, 16). Budding of RuV occurs at the Golgi complex, and the viral particles then transit through the secretory pathway and are released extracellularly (17, 18). Expression of the structural proteins alone produces virus-like particles that bud into the Golgi (15, 18, 19).

The budding process of RuV is poorly understood. E2 and E1 form heterodimers within the ER and then transit to the Golgi (11, 21). Transport of either membrane protein alone is inefficient, and the targeting of the heterodimer to the Golgi is mediated by a Golgi targeting/retention sequence that maps to the E2 TM domain (11, 21, 22). Cp is found in homodimers both in infected cells and viral particles (23). Expression of Cp in the absence of the envelope proteins produces a reticular pattern consistent with ER association through the Cp TM domain (14), while expression in the complete polyprotein leads to Cp localization in the Golgi (14, 20). While localization of E2, E1, and Cp to the Golgi is dependent on the Golgi targeting sequence in the E2 TM domain, the Cp interactions required are unclear, and have been suggested to involve the Cp TM domain and the E2 protein or E2/E1 heterodimer (14, 24). Alternatively, the E2 arginine-rich endodomain region (Fig. 1A) has been suggested to interact with an acidic region on the Cp (25), and mutation of several of these E2 residues to alanine inhibited particle release (18). Once RuV particles bud into the Golgi lumen, the E1 TM and cytoplasmic tail are reported to play a role in particle secretion (18, 26).

A number of enveloped viruses have evolved to use the cellular ESCRT (endosomal sorting complex required for transport) machinery for budding (27). The ESCRT pathway mediates the fission of membranes that bud topologically away from the cytoplasm, such as the budding of vesicles into the lumen of multivesicular bodies (28). Viruses can encode short peptide motifs known as late domains in their structural proteins. Such late domains act to recruit the ESCRT machinery to the budding site (27, 29). The ESCRT-dependence of virus budding can be tested by expression of a dominant-negative variant of VPS4, the AAA+ATPase that acts to disassemble the ESCRT-III complex at the budding site (27, 29, 30). Using this method along with detailed analyses of late domains, budding of viruses such as retroviruses, filoviruses, and rhabdoviruses has been shown to be ESCRT-dependent. In contrast, viruses, including alphaviruses and influenza A virus, have been shown to be ESCRT-independent (27). The potential role of the ESCRT complex in RuV budding has not been addressed.

Through bioinformatic motif mining, we identified a potential late domain motif, ^278^PPAY^281^, at the C-terminus of E2. Mutation of E2 Y281 to A reduced RuV growth by >3 logs. However, RuV production was insensitive to the expression of dominant-negative VPS4A, suggesting that RuV budding was ESCRT-independent. Analysis of virus-infected cells showed that structural protein synthesis, processing, and localization of E2 and E1 were comparable between WT and mutant. However, unlike the WT, the mutant E2E1 did not pulldown Cp, and in mutant-infected cells, Cp did not colocalize with the envelope proteins and was not targeted to the Golgi complex. Two revertants of E2 Y281A increased virus growth, particle production, Cp-E2E1 interaction, and Cp Golgi targeting. Analysis of the rescue mutations demonstrated the close evolutionary relationship between the human-hosted RuV and bat-hosted RuhV, and provided genetic evidence for E2-Cp interaction.

## RESULTS

### Identification of short linear motifs in the RuV structural proteins

Short linear motifs (SLiMs) are short motifs of 3–15 residues in eukaryotic proteins that facilitate protein-protein interactions in diverse cellular processes. Viruses and other pathogens can also encode SLiM in their proteome to imitate such interactions and thus repurpose host cell machinery (31). To identify SLiMs and potential host factors involved in the RuV egress process, the RuV structural protein ORF (1,063 aa) was analyzed using the Eukaryotic Linear Motif server (32). Our computational analysis identified >350 SLiMs in the RuV structural ORF, including several potential late domain motifs. Such late-domain motifs can promote virus budding by recruiting the cellular ESCRT machinery to the budding site (27, 33). We then evaluated these motifs in the context of the proposed topology of the RuV structural proteins (Fig. 1A) and the published literature.

### A potential late domain motif at the E2 C-terminus that affects RuV production

Our bioinformatic analyses identified three canonical late domain motifs in the RuV structural proteins (Fig. 1A). Two are on the E1 ectodomain (E1 ^67^PTAP^70^ and ^394^PPPY^397^) and would not be accessible to the ESCRT machinery in the cytoplasm (27, 32). The E2 ^278^PPAY^281^ motif is located at the E2 C-terminal region, which serves as the E1 signal sequence and is proposed to form a second E2 TM domain. However, the TM nature of this region has not been confirmed. If this region is released from the membrane after its signal sequence function, the E2 ^278^PPAY^281^ motif would then be available to recruit the ESCRT machinery. We tested the role of this motif in virus exit by generating E2 P278A and E2 Y281A mutations in the RuV infectious clone (Fig. 1B). Such alanine substitutions are commonly used to confirm viral late domains, as in the example of vesicular stomatitis virus (VSV), an ESCRT-dependent virus containing a PPPY motif (30, 34). WT and mutant RuV RNAs were prepared by *in vitro* transcription and electroporated into BHK-21 cells, and the time course of production of infectious virus was evaluated for duplicate clones (Fig. 1C). Production of the E2 P278A mutant was about 0.5 logs lower than that of the WT virus. In contrast, production of the E2 Y281A mutant was not detectable at early time points, and levels were reduced by about 3 logs at the 48 h time point (Fig. 1C). To determine if the reduction in virus titer reflected a reduction in virus particle production, media from WT and mutant-infected cells were collected 48 h post-electroporation, pelleted through a sucrose cushion, and analyzed by SDS-PAGE and western blotting (Fig. 1D). Particle production was decreased but clearly detectable for the E2 P278A mutant, in keeping with its relatively modest decrease in growth. In contrast, little Cp was detected in the pelleted virus fraction for E2 Y281A. While small amounts of E1 protein were detected, this may reflect membrane fragments, particularly since the E1/Cp ratio was ~5× higher than that in the WT and E2 P278A pellets. While RuV buds into the Golgi, its envelope glycoproteins have been shown to transit to the cell surface (35, 36). Analysis of lysates of the WT and mutant-infected cells confirmed the expression of all of the structural proteins. Expression was somewhat reduced in the E2 Y281A-infected cells, presumably due to the strong decrease in infectious particle yield and thus decreased secondary infection. Taken together, our data indicate that the E2 Y281A mutation caused a strong effect on RuV production.

### Expression of dominant-negative VPS4A does not inhibit RuV production

To test the role of the ESCRT machinery in RuV exit, we used T-REx-U-2 OS cells to generate clonal cell lines that inducibly express GFP-tagged VPS4A-WT or the VPS4A-EQ mutant lacking ATPase activity. Expression of the inactive form of either the VPS4A or VPS4B paralogs (29) blocks the ESCRT pathway (30, 33) and has been extensively used as a standard experimental technique to interrogate the ESCRT dependence of different viruses (29, 30, 33). As previously observed for other cell lines that inducibly express VPS4EQ (37), expression of GFP-VPS4A-EQ in U2-OS cells produced a GFP-labeled vesicular compartment that represents enlarged endosomes (Fig. S1). To confirm the inhibitory effect of VPS4-EQ expression in our clonal cell lines, we tested the production of the ESCRT-dependent VSV. As we previously observed in HEK293 cells (30), VSV production from U2-OS cells was reduced ~15-fold by expression of VPS4A-EQ, but was not significantly affected by the expression of the WT VPS4A (Table 1). To test the effect on RuV production, cells were infected at an MOI of 15 IC/cell and incubated for 29 h, a time point chosen to establish strong virus infection and facilitate detection of subsequent RuV production. VPS4 expression was then induced and media samples were collected after 1 or 13 h further incubation. The results show that RuV production was unaffected by the expression of either VPS4A-WT or VPS4A-EQ (Table 1), suggesting that RuV budding was not ESCRT-dependent. We therefore explored other features of RuV biogenesis that might explain the deleterious effect of the E2 Y281A mutation.

**TABLE 1:** Effect of VSP4A-EQ expression on virus budding

		RuV		VSV	
T-REx cell line	Induction status	Time post-treatment (h)*^a^*	Titer (IC/mL)^*c*^ (fold change^*d*^)	Time post-treatment (h)*^b^*	Titer (PFU/mL)^*c*^ (fold change^*d*^)
VPS4A-EQ	Uninduced	1	ND	2.2	7.0 × 10^2^
	Induced	1	1.5 × 10^2^	2.2	8.3 × 10^2^
	Uninduced	13	4.4 × 10^4^	8	1.2 × 10^6^
	Induced	13	3.1 × 10^4^ (1.3 ± 0.2)	8	1.0 × 10^5^ (14.6 ± 4.4)
VPS4A-WT	Uninduced	1	ND	2.2	8.0 × 10^2^
	Induced	1	1.0 × 10^2^	2.2	1.3 × 10^3^
	Uninduced	13	4.5 × 10^4^	8	3.3 × 10^5^
	Induced	13	5.0 × 10^4^ (1.0 ± 0.07)	8	5.0 × 10^5^ (1.6 ± 0.5)

^*a*^ VPS4A-EQ/WT cells were infected with RuV (MOI 15 IC/cell) for 4 h, washed and incubated for 29 h. Cells were then washed 3× and treated with or without 1 µg/mL doxycycline. Media were collected for titration at 1 or 13 h post-treatment.

^*b*^ VPS4A-EQ/WT cells were treated with or without 1 µg/mL doxycycline for 1 h and then infected with VSV at 10 PFU/cell for 1 h. Cells were then washed 3× and media were collected for titration at 2.2 or 8 h post-treatment.

^*c*^ Titers shown are data from one of three independent experiments. ND = not detected.

^*d*^ Fold change calculated as (titer of uninduced/induced). The average with standard deviation of three to four independent experiments is shown.

### Synthesis and processing of RuV WT and mutant structural proteins

**Fig 2:**
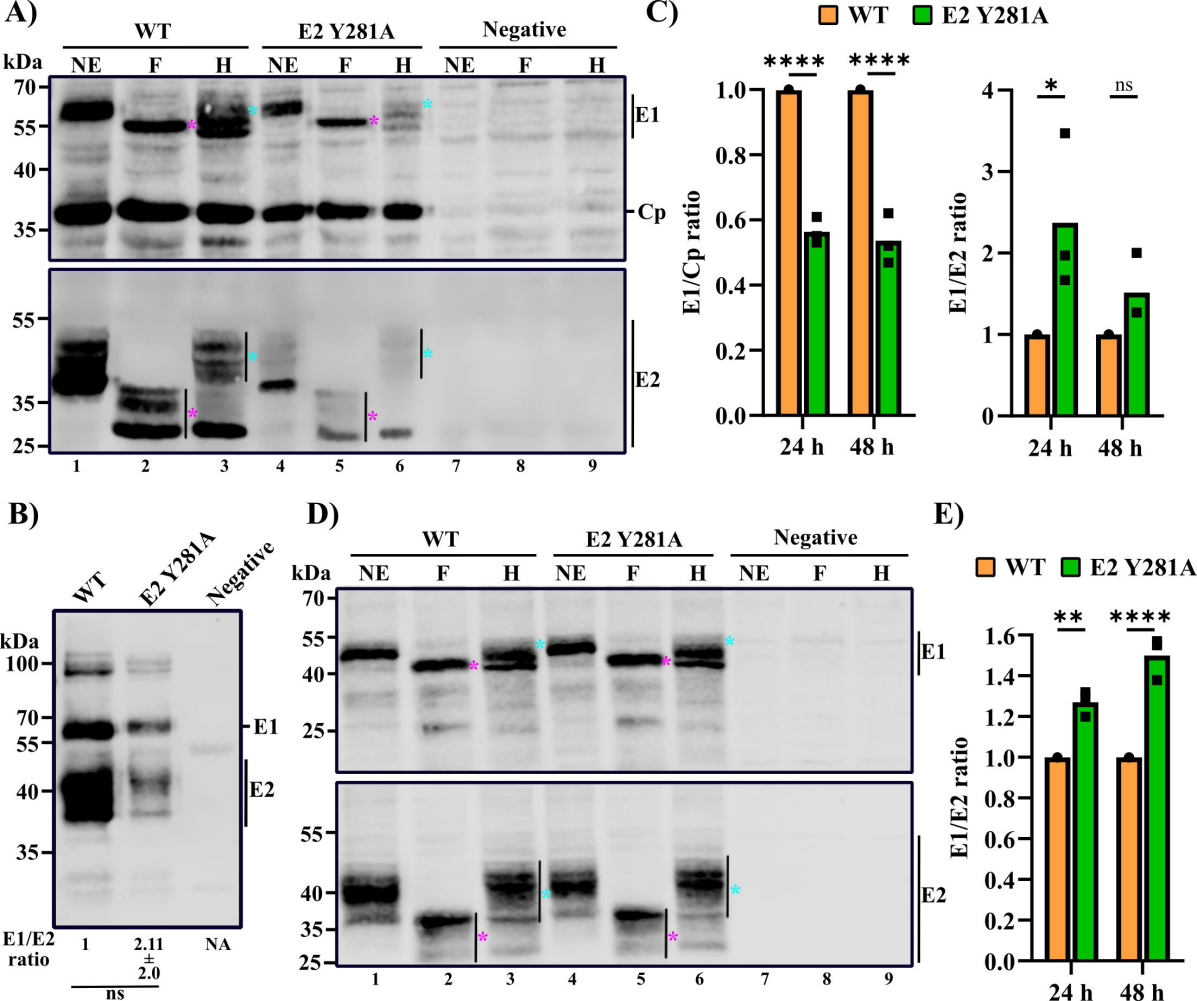
Characterization of biogenesis of the E2 Y281A structural proteins. (**A–C**) BHK-21 cells were electroporated with WT or E2 Y281A viral RNA, cultured to allow viral protein production, lysed, and analyzed as indicated. Negative indicates no RNA. (**A**) Processing and glycosylation of WT and E2 Y281A structural polyproteins. Lysates at 42 h post-electroporation were treated with no enzyme (NE), PNGaseF (**F**) or EndoH (**H**) and analyzed by SDS-PAGE and western blotting with RuV pAb (top panel) or E2 mAb (bottom panel). The PNGaseF-sensitive (magenta star) and EndoH-resistant (cyan star) forms of E1 and E2 are indicated. (**B**) E2/E1 heterodimer formation. Lysates at 42 h post-electroporation were immunoprecipitated with E1 mAb. Co-immunoprecipitation of E2 was analyzed by SDS-PAGE and western blotting with RuV pAb. Note that the E1 mAb was used for co-IP analysis, as the E2 mAb was active in WB but not IP. (**C**) E1/Cp or E1/E2 ratio of 24 or 48 h lysate quantified from western blot with RuV pAb and E2 mAb. (**D and E**) Vero cells were transfected with expression plasmids encoding WT or E2 Y281A E2/E1, without Cp. (**D**) Cells were cultured for 48 h, lysed, and analyzed as in (A). Negative indicates no plasmid control. (**E**) Cells were cultured for 24 or 48 h post-transfection. The E1/E2 ratio in the cell lysates was determined by western blot with RuV pAb and E2 mAb. For (C and E), the data are the average of three independent experiments that are normalized to the WT E1/Cp or E1/E2 ratio, set as 1.0. Panels (A and B) are representative examples of three independent experiments. Statistical analyses were carried out by unpaired *t* test (**B**) and two-way ANOVA with Sidak multiple comparisons tests (**C and E**). ****, *P* < 0.0001; **, *P* < 0.01; *, *P* < 0.05. NA: not applicable.

To characterize the biogenesis of the RuV structural proteins, we electroporated BHK-21 cells with WT or E2 Y281A viral RNAs and cultured for 42 h. The cells were then lysed and treated as indicated with PNGaseF or EndoH, and analyzed by SDS-PAGE and western blotting (Fig. 2A). E2 and E1 have predicted molecular weights of 31 kDa (~42–47 kDa after glycosylation) and 51 kDa (~58 kDa after glycosylation), respectively. The results showed that both WT and mutant E2 and E1 had similar migration patterns in SDS-PAGE, and acquired N-linked carbohydrates as reflected by their sensitivity to PNGase F digestion. A similar proportion of E2 and E1 were EndoH-resistant in WT- versus mutant-infected cells, suggesting comparable formation of E2-E1 heterodimers in the ER and comparable transport to the Golgi compartment (21). To directly assay for the formation of the E2-E1 heterodimer, lysates of WT and mutant-infected cells 42 h post-electroporation were immunoprecipitated with an E1 monoclonal antibody (mAb) and analyzed by SDS-PAGE and western blotting with RuV polyclonal serum that recognizes both E2 and E1. The results of these co-IP experiments indicated comparable E2-E1 heterodimer formation for WT and E2 Y281A (Fig. 2B). Thus, while the viral protein amounts differed between WT and E2 Y281A-infected cells due to reduced secondary infection by the mutant, the qualitative features of the proteins appeared similar.

Comparison of the ratios of the structural proteins in WT- and mutant-infected cells at 24 and 48 h post-electroporation showed that the mutant had a lower ratio of E1/Cp and a somewhat higher ratio of E1/E2 compared to the WT (Fig. 2C). To determine if this might be due to the observed decreases in protein expression and/or virus budding in mutant-infected cells, we also analyzed Vero cells transfected with plasmids expressing only the E2/E1 proteins from the WT or E2 Y281A mutant. The glycosylation profiles of the envelope proteins were similar between WT and the E2 Y281A mutant (Fig. 2D). Comparison of the steady-state levels of the envelope proteins at 24 and 48 h post-transfection showed that the ratio of E1/E2 was somewhat higher for the mutant (Fig. 2E). However, the relatively modest decreases in the level of E2 appeared insufficient to cause the observed 3 log decrease in particle production.

### Intracellular localization of the WT and mutant structural proteins

**Fig 3:**
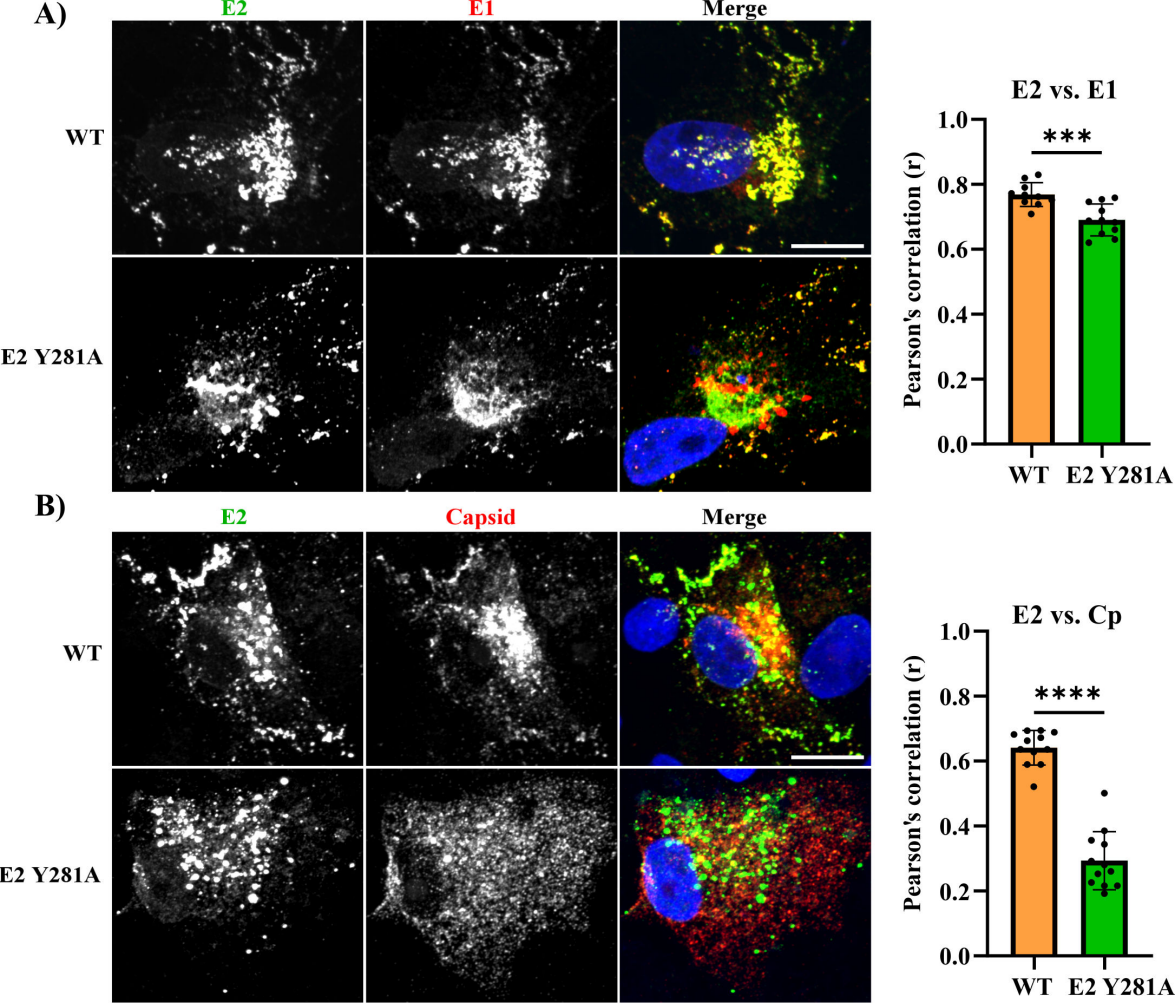
Localization of WT and mutant structural proteins. Vero cells were transfected with RuV WT or E2 Y281A RNA. At 42 h post-transfection, the cells were fixed, permeabilized, and stained with Hoechst to visualize the nuclei and with mAbs to (**A**) E2 and E1, or (**B**) E2 and Cp. Samples were imaged by confocal microscopy and images are representative examples from two independent experiments. The bar graph in each panel shows the Pearson’s correlation coefficient (r) determined for 10–15 cells/sample from one experiment, showing the individual data points and the mean and standard deviation. Statistical analyses were carried out by unpaired *t* test. ****, *P* < 0.0001; ***, *P* < 0.001. Scale bar = 10 µm.

**Fig 4:**
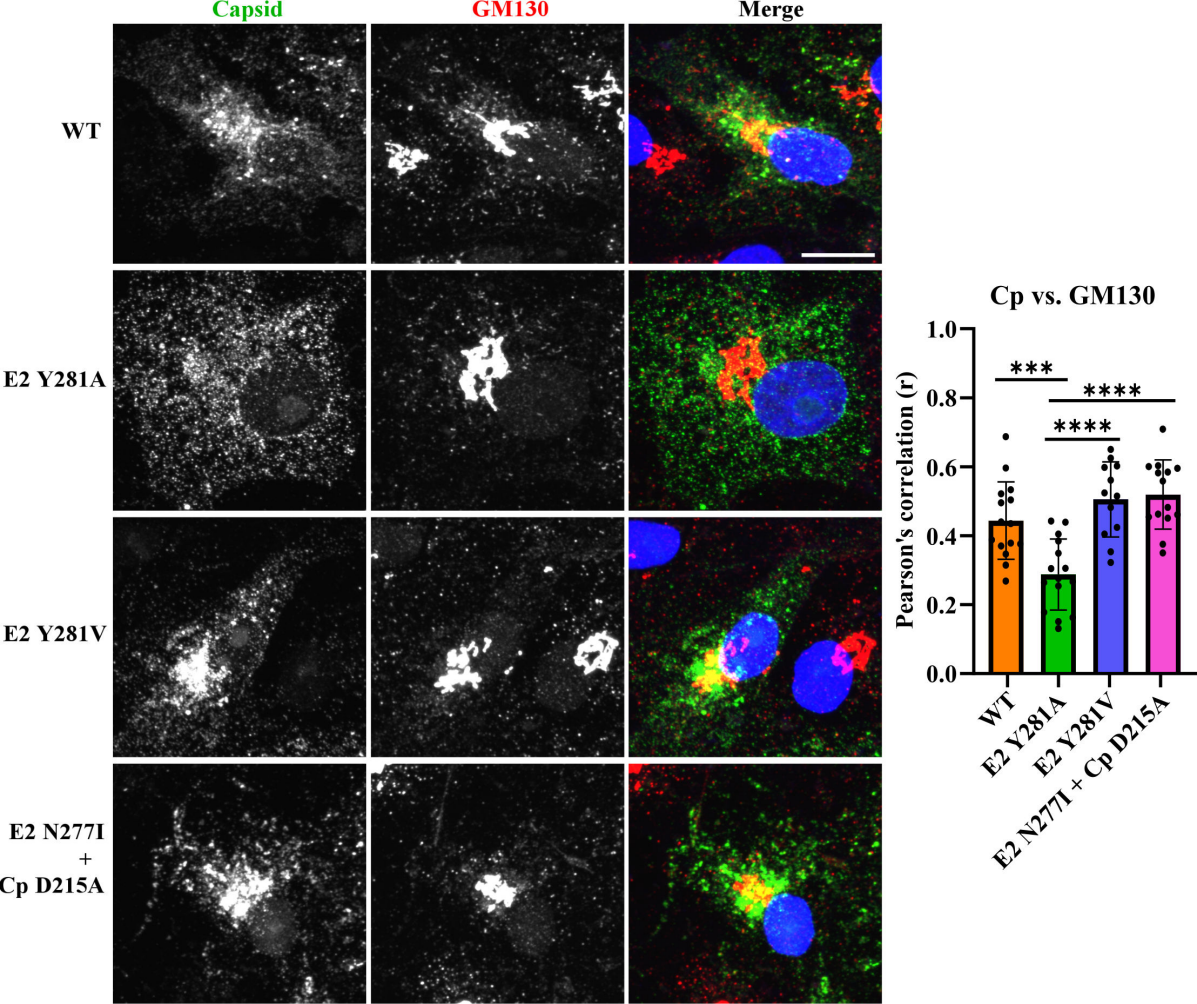
Localization of Cp and the Golgi marker GM130. Vero cells were transfected with RNA from RuV WT or the indicated mutant or revertant. At 42 h post-transfection, the cells were fixed, permeabilized, and stained with mAbs to Cp and GM130, and with Hoechst to visualize the nuclei. The localization of Cp and the Golgi marker GM130 was evaluated by confocal imaging as in Fig. 3. Images are a representative example of two independent experiments. The bar graph in the right panel shows the Pearson’s correlation coefficient (r) determined for 10–15 cells/sample from one experiment, showing the individual data points and the mean and standard deviation. Statistical analyses were carried out by one-way ANOVA with Dunnett’s multiple comparisons test. ****, *P* < 0.0001; ***, *P* < 0.001. Scale bar = 10 µm.

We next examined the intracellular localization of the structural proteins in WT- and E2 Y281A-infected cells. Vero cells were transfected with WT or mutant viral RNAs, fixed at 42 h post-transfection, stained with mAbs against the RuV structural proteins, and imaged by confocal microscopy. In keeping with the co-IP studies, the E2 and E1 glycoproteins colocalized in the perinuclear region of both WT and mutant-infected cells, although this colocalization was stronger for the WT than for the E2 Y281A mutant (Fig. 3A). In contrast, while both E2 and E1 strongly co-localized with Cp in the perinuclear region of WT-infected cells as previously reported (24), the Cp in mutant-infected cells was diffusely distributed in the cytoplasm (Fig. 3B; Fig. S2). Quantitation of E2-Cp colocalization revealed a striking difference in the Pearson’s correlation coefficient (*r*) between the two samples (WT 0.7 vs mutant 0.3). Similar differences were observed for E1-Cp colocalization (*r* values WT 0.7 vs mutant 0.5) (Fig. S2). While the Cp strongly co-localized with the Golgi marker GM130 in WT-infected cells (*r* = 0.5), this colocalization was significantly decreased in the mutant-infected cells (*r* = 0.3) (Fig. 4). Thus the E2 Y281A mutation disrupts Cp transport to the Golgi, the site of RuV budding.

### Isolation and characterization of E2 Y281A revertants

**Fig 5:**
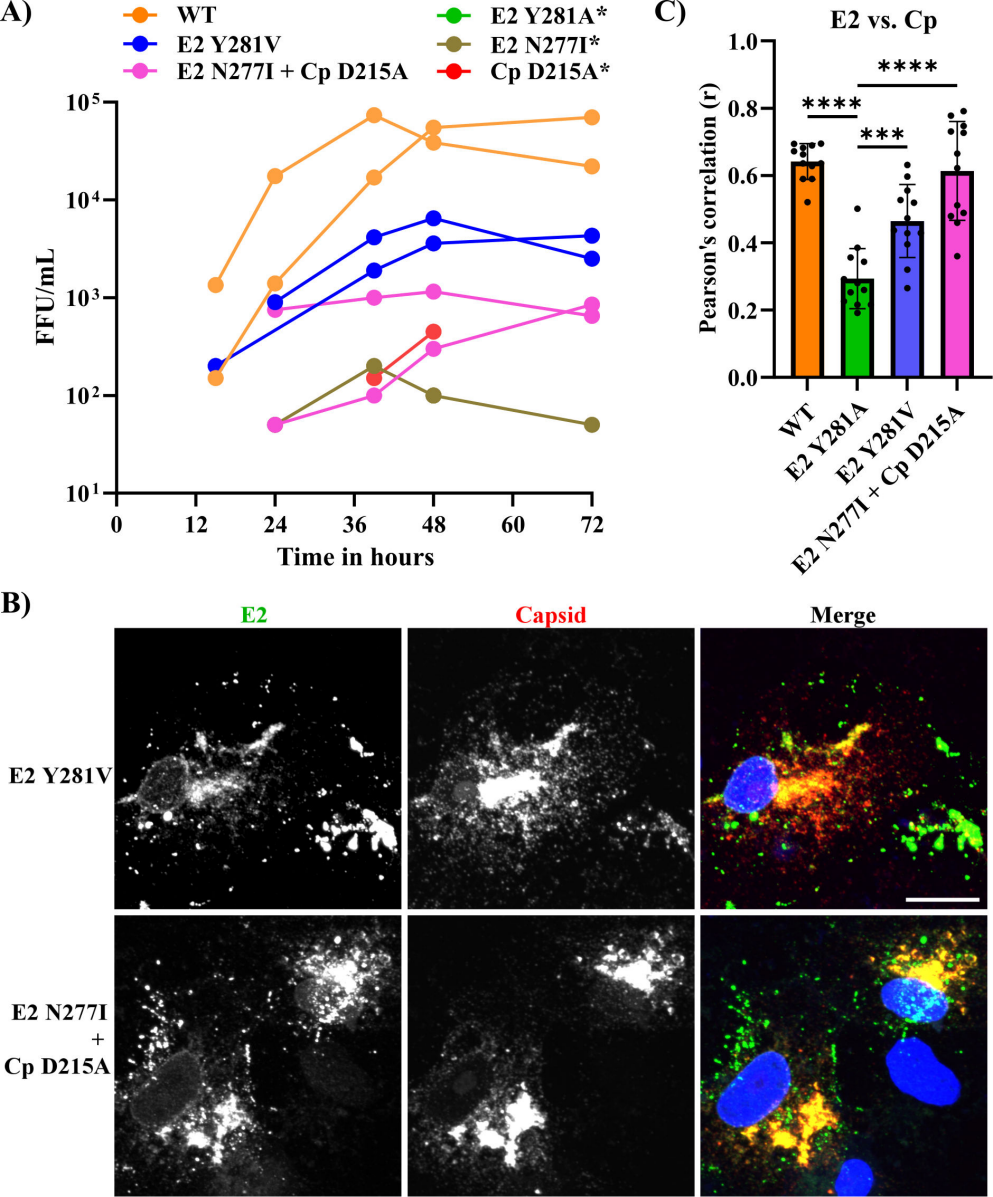
Growth and structural protein localization of E2 Y281A revertants. (**A**) Growth kinetics of WT RuV, E2 Y281A, and the indicated revertant constructs. *In vitro* transcribed RNAs were electroporated as in Fig. 1C and progeny virus production was quantitated by focus forming assay. Graphs show individual data from two independent experiments at the indicated time points. * indicates that virus was not detected from one or both of the experiments. (**B**) Analysis of E2 and Cp protein localization for the indicated E2 Y281A revertant constructs was performed as in Fig. 3B. Images are a representative example from two independent experiments. (**C**) Pearson’s correlation coefficient (r) analysis of the E2/Cp localization data were performed as in Fig. 3B. Statistical analyses were carried out by one-way ANOVA with Dunnett’s multiple comparisons test. ****, *P* < 0.0001; ***, *P* < 0.001. Scale bar = 10 µm.

We passaged the E2 Y281A mutant on Vero cells to select for rescue of its impaired growth. We then isolated candidate revertants by serial dilution, and analyzed the sequence of their structural protein regions. Two revertants were identified: (i) the pseudorevertant E2 281V, which was independently isolated four times, and (ii) a second-site revertant, [E2 N277I + Cp D215A] (Table 2). To assess the effects of these mutations, we engineered them individually or in combination into the pBRM33 Y281A RuV infectious clone and electroporated the viral RNAs into BHK-21 cells to test for effects on growth (Fig. 5A). Substitution of E2 281A by E2 281V significantly rescued virus growth. The growth of E2 Y281A was also increased by the addition of the second site mutations [E2 N277I + Cp D215A]. No reproducible increase in E2 Y281A growth was observed from the inclusion of E2 N277I or Cp D215A alone in the E2 Y281A background, indicating that both mutations were needed for rescue. While both the E2 Y281V and [E2 N277I + Cp D215A] mutations conferred improved virus growth on the E2 Y281A mutant, they did not completely rescue the mutant growth kinetics (Fig. 5A). Immunofluorescence analysis showed that the revertants display increased colocalization of Cp with E2 or E1 (Fig. 5B and C; Fig. S2). Colocalization of Cp with the Golgi marker GM130 was also increased in the revertants (Fig. 4). Thus, for both revertants there is a strong correlation between increased growth and increased Cp localization with the envelope proteins in the Golgi.

**TABLE 2:** Revertants of RuV E2 Y281A mutant^*a*^

Revertant	WT codon	WT residue	Revertant codon	Revertant residue	No. of independent isolates
E2 281V	TAT	Y	GTG	V	4
E2 277I +Cp 215A	AAC	N	ATC	I	1
	GAC	D	GCC	A	

^*a*^ The mutant E2 sequence was GCG (A) versus WT TAT (Y). Note that the revertants were either pseudorevertants (E2 281V) or second-site revertants (E2 277I + Cp 215A), but will be referred to collectively as revertants.

We analyzed the ratios of the revertant structural proteins in electroporated BHK-21 cell lysates and pelleted viruses (Fig. S3). The intracellular E1/E2 and E1/Cp ratios were not significantly different between the WT, Y281A, or revertants. In the pelleted samples, the ratios of E1/E2 were similar among the WT, Y281A, and revertants. However, the E1/Cp ratios in the revertant virus pellets were comparable to those of WT and significantly lower than that of E2 Y281A, indicating the rescue of production of revertant particles containing Cp. The particle yield was higher for E2 Y281V than for the second-site revertant [E2 N277I + Cp D215A], in keeping with its increased growth kinetics.

### Interaction of capsid and glycoproteins

**Fig 6:**
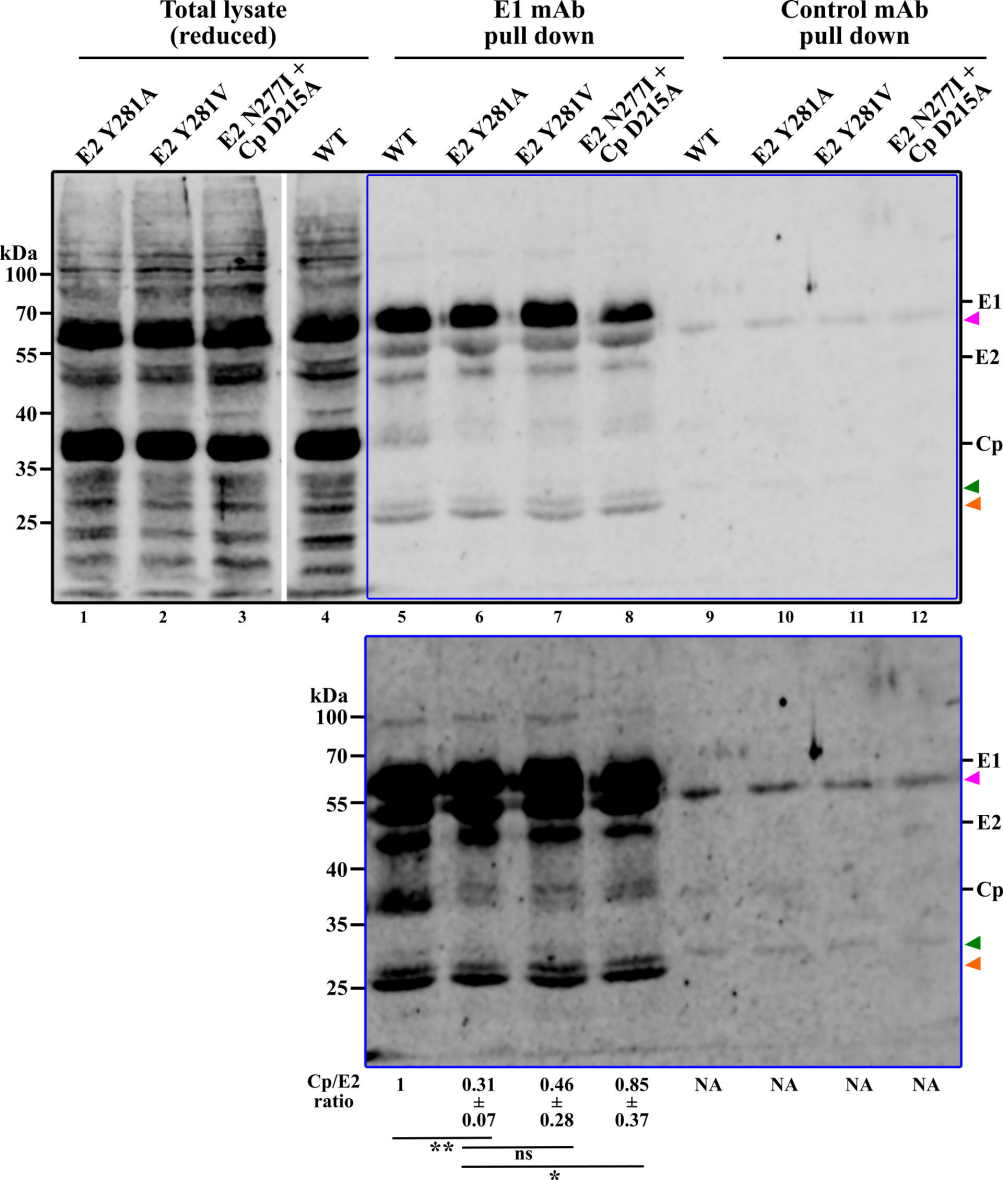
Co-immunoprecipitation of Cp and envelope proteins. BHK-21 cells were electroporated with WT or mutant RuV RNAs. At 42 h post-electroporation, the cells were crosslinked with DSP and quenched. Cell lysates were immunoprecipitated by mAb to E1 or control mAb, the crosslinker reversed by reduction, and the samples analyzed by western blotting with RuV pAb. The white space between lanes 3 and 4 indicates where two separate blot images from the same experiment have been combined. The mAb heavy and light chains are indicated by the magenta and green arrowheads, respectively. The orange arrowhead indicates what is likely a non-glycosylated form of E2. The area indicated by the blue box in the top panel is shown with enhanced contrast in the bottom panel. The mean Cp/E2 ratio ±standard deviation (*n* = 3–4) is indicated under each lane, and was normalized to the WT, set as 1. Statistical analyses were carried out by one-way ANOVA with Dunnett’s multiple comparisons test. **, *P* < 0.01; *, *P* < 0.05. NA: not applicable.

The requirement for the mutations in both E2 and Cp in the second-site revertant suggested that they act synergistically to rescue a Cp-E2 interaction disrupted by the E2 Y281A mutation. Since our previous E1-mediated E2 pull-down did not retrieve Cp (Fig. 2B), we reasoned that such a Cp-E2 interaction might be labile under conditions of lysis and IP. Therefore, prior to co-IP, potential Cp-E2 interactions in infected cells were stabilized using DSP, a thiol-cleavable primary amine crosslinker. Lysates were then immunoprecipitated with a mAb to E1 or a control mAb, reduced to reverse the crosslink, and analyzed by SDS-PAGE and western blot using RuV pAb to identify Cp, E2 and E1 (Fig. 6). The WT, mutant and revertant samples all showed efficient retrieval of E2 as expected. IP of the WT E2/E1 retrieved Cp relatively efficiently (Fig. 6). In contrast, the E2 Y281A sample showed significantly reduced Cp retrieval. We quantitated Cp retrieval by determining the Cp/E2 ratio for all of the IP samples, setting the WT ratio to 1. The Cp/E2 ratio was 0.31 for E2 Y281A, 0.46 for the pseudorevertant E2 Y281V and 0.85 for the second-site revertant [E2 N277I + Cp D215A] (Fig. 6). Thus, glycoprotein-mediated Cp retrieval was quantitatively increased in the revertants versus the E2 Y281A mutant. Our mAb E1 pull-down experiments thus showed that the E2 Y281A mutation disrupts the Cp-E2 interaction while the revertants at least partially rescue this interaction.

### Bioinformatic analysis of the RuV E2 C-terminus

**Fig 7:**
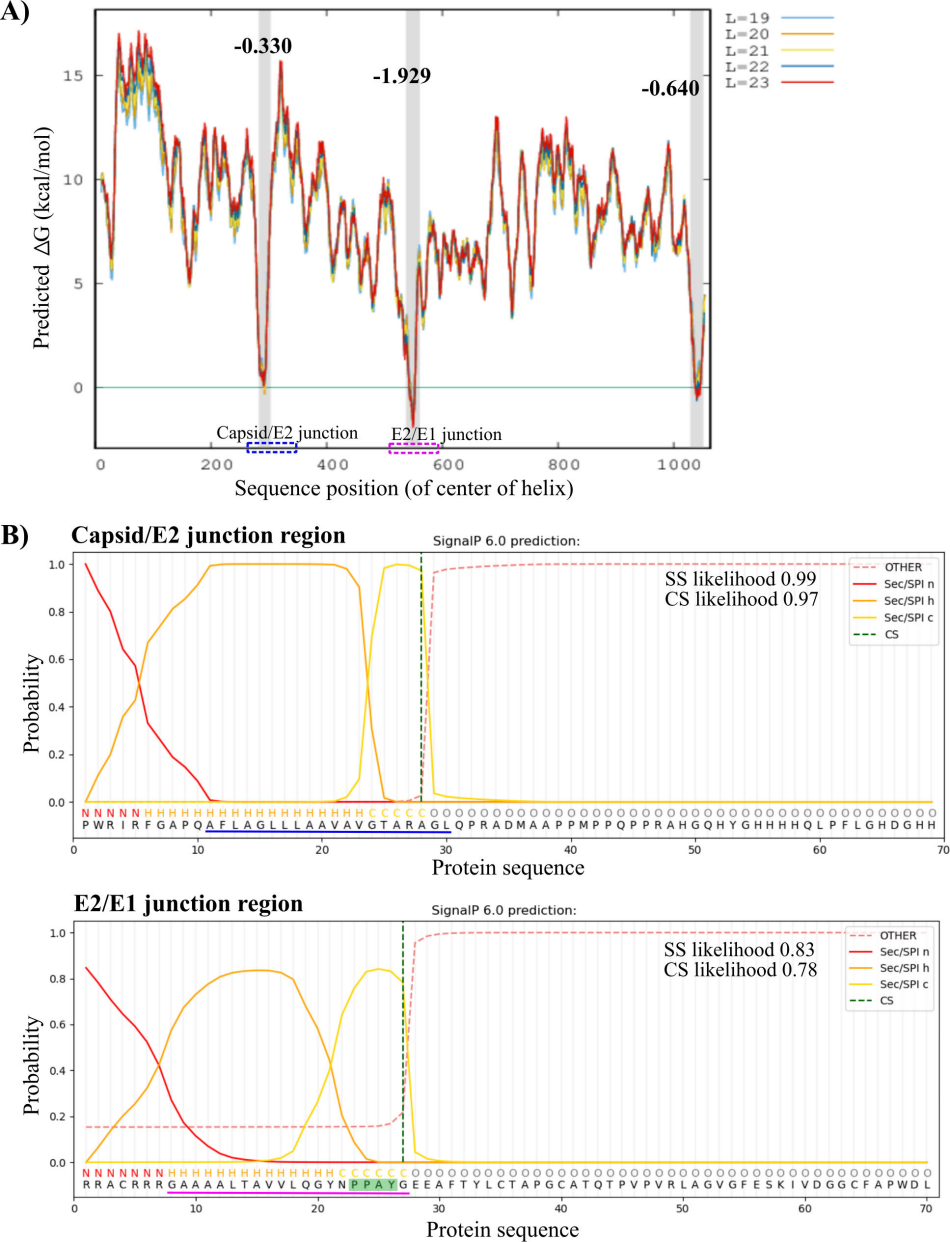
Prediction of transmembrane domains, signal sequences, and signal peptidase cleavage sites. (**A**) The RuV structural polyprotein was scanned (39) for TM helices of lengths from 19 to 23 residues, as indicated by the colors at the right. Three regions of the polyprotein: 283–302 (Cp^283–300^), 538–560 (E2^238–260^), and 1029–1050 (E1^447–468^) were identified as having negative Δ*G* values, supportive of their serving as TM domains (highlighted in gray with their corresponding Δ*G* values indicated). Additionally, TOPCONS analysis (41) predicted that the polyprotein regions 280–300, 535–555, and 1031–1051 were strongly favored to serve as TM domains. Neither analysis predicted that the E2 C-terminus (residues 563–582) was a TM domain. The Cp/E2 and E2/E1 junction regions that are shown at the sequence level in (B) are highlighted with blue and magenta dotted boxes, respectively. (**B**) Signal sequences (SS) were predicted using SignalP-6.0 (43) by analyzing the 70 aa junction regions including the approximate cleavage sites (CS) between Cp/E2 (upper panel) and E2/E1 (lower panel). The N-terminal (N in red), hydrophobic center (H in dark yellow) and C-terminal (C in light yellow) residues of the predicted SS are indicated above the sequence and by a probability line of corresponding color. The blue line under the sequence in the upper panel indicates the predicted TM helix, while the magenta line under the sequence containing the PPAY motif (green shading) in the lower panel indicates the region not predicted to be a TM helix (based on the analysis in Table 3; Fig. 7A). The predicted C-terminal residue generated by signal peptidase cleavage is marked by a dotted green line. Sequences that are not predicted to be part of the SS are marked by O (other) and a dotted red line. The likelihood of SS and CS is shown for each region by the added text.

Prior literature (2, 11, 15, 18, 25) posited that the E1SS comprising E2 residues ~263–282 remains membrane-embedded, as diagramed in the topology model in Fig. 1A. However, our co-IP and second-site revertant results provided both biochemical and genetic evidence for a Cp-E2 endodomain interaction that rescued both Cp transport to the Golgi and virus production. We therefore analyzed the propensity for the E1SS to remain membrane-inserted. The complete RuV structural protein ORF was processed through the ΔG prediction server (38, 39). A negative ΔG value indicates a strong probability that a sequence would be transferred into the ER membrane by the Sec61 translocon and serve as a TM helix (38–40). Our analysis identified three regions in the RuV structural proteins with negative ΔG values: E2SS (Cp^283–300^), E2TM (E2^238–260^) and E1TM (E1^447–468^) (Table 3; Fig. 7A). In contrast, the ΔG value of the E1SS (E2^263–282^) was strongly positive, 4.965 kcal/mol, suggesting that this region including E2 Y281 does not serve as a TM domain. Further analysis using the TOPCONS (41) server predicted almost identical regions of the structural proteins as TM domains. Analysis of the E2 sequence using Phyre2 (42) also predicted that the E2TM but not the E1SS remains membrane-anchored. We used the SignalP-6.0 server (43) to analyze the regions encompassing the Cp-E2 and E2-E1 junctions (Table 3; Fig. 7B). Both of these regions were predicted to contain SS and signal peptide cleavage sites with a high degree of confidence, but only the E2SS was predicted to serve as a TM domain. Our analyses are thus consistent with previous experimental reports that the E2SS serves as a membrane anchor for the Cp (13, 24), but they indicate that the E1SS is not a TM domain.

**TABLE 3:** Predicted TM and SS in RuV structural proteins

Amino acid sequence	Protein	Predicted ΔG as TM domain	SS likelihood*^b^*
^283^AFLAGLLLAAVAVGTARA^300^-*GL^a^*	Capsid	−0.330 (0.010^*c*^)	0.99
^238^AFAAFVLLVPWVLIFMVCRRACR^260^	E2	−1.929	0
^263^GAAAALTAVVLQGYNPPAYG^282^	E2	4.965	0.83
^447^WWQLTLGAICAPLLAGLLACCA^468^	E1	−0.640	0

^*a*^ The last two residues *GL* are the extreme N-terminal aa of E2.

^*b*^ See Fig. 7B for SS (signal sequence) prediction.

^*c*^ ΔG without *GL.*

### Sequence comparisons of RuV with the novel rubiviruses

**Fig 8:**
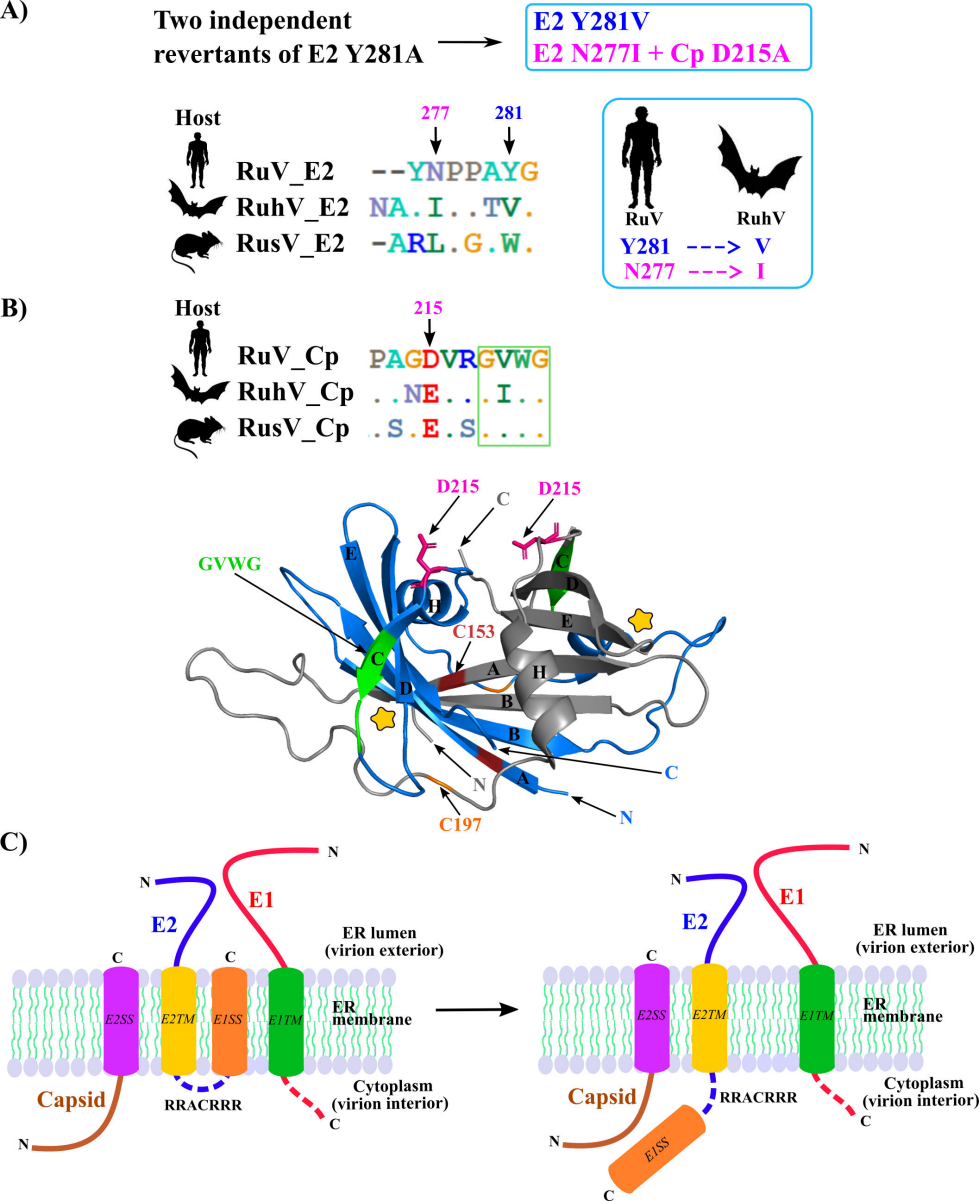
Revertant evolutionary and structural features. (**A**) E2 Y281A revertant sequences compared to sequences of other rubiviruses. The upper panel (cyan box) shows the revertant sequences in dark blue and magenta. The lower panel shows the sequence alignment of the E2 281 regions of RuV (gene bank ID P08563), RuhV (gene bank ID QKO01647.1), and RusV (gene bank ID QKO01649.2), with arrows indicating RuV positions 281 (blue) and 277 (magenta). The cyan box on the right highlights the functional replacement of the RuV residue by the corresponding RuhV residue. (**B**) Upper panel: the second-site revertant also contained an alanine in the Cp 215 position (magenta), which the sequence alignment using the reference sequence as in panel (A) shows is conserved as an acidic residue among the three rubiviruses. Lower panel: RuV capsid structure prepared using PDB file 4HAR (25) and the PyMOL program (44). Capsid monomers (colored blue or gray) form the antiparallel Cp dimer. The N and C termini are indicated in blue or gray for each monomer, and the A-E β-strands of each monomer and the H helix are labeled in black letters. The positions of the residues involved in disulfide bonding (C153 in rosewood and C197 in orange) are indicated for one monomer and highlighted for both. The GVWG motif involved in inter-dimer interaction (25) is indicated for one monomer (in green) and highlighted in green for both (see also the green box in Cp sequence in upper panel). The Cp pocket previously hypothesized to bind the E2 arginine-rich region (25) is indicated by the yellow star on each Cp monomer. D215 is shown in a magenta stick representation on each monomer. Note that the D215 residues are located close to the ^218^GVWG^221^ sequence and are on the same side of the Cp dimer. (**C**) Model for the rearrangement of the RuV E2 protein on the ER membrane. Coloring of protein regions as in Fig. 1A. The E1SS at the E2 C-terminus was thought to remain inside the membrane (left panel), but our experimental and bioinformatic analyses suggest that this E2 region reorients towards the cytoplasm where it can interact with capsid protein (right panel).

The overall amino acid sequence of the RuV structural proteins is ~51 and ~41% similar to that of RuhV and RusV, respectively (7). Alignment of the E2 C-terminal regions of these three viruses revealed that although E2 Pro 278 is conserved, the PPAY motif is not (Fig. 8A). This lack of sequence conservation provides support to our finding that RuV budding is ESCRT-independent. In addition, SLiM analysis of RusV and RuhV did not reveal any canonical late domain motifs in their envelope glycoproteins. Comparison of the C-terminal sequence of RuV E2 with the sequences of the analogous regions in RuhV and RusV shows that RuV E2 Y281 is a V in RuhV, and RuV E2 277N is I in RuhV and L in RusV (Fig. 8A). Strikingly, selection for growth of the E2 Y281A mutant produced RuV revertants containing either E2 281V or E2 277I. Thus these rescue mutations support the close evolutionary relationship between the human-hosted RuV and bat-hosted RuhV.

In addition to E2 N277I, rescue in the second-site revertant also required the Cp D215A mutation. This Cp residue is conserved as an acidic amino acid across these three rubiviruses (Fig. 8B). The RuV Cp forms a homodimer composed of two antiparallel Cp monomers that can be stabilized by disulfide linkage (25) (Fig. 8B). The Cp dimer structure indicates that both of the D215 residues are present at one side of the dimer, and are located close to a ^218^GVWG^221^ motif. This Cp motif is strongly conserved and is implicated in interdimer interaction (25). Using the NCBI Virus website we expanded our sequence analysis to align >100 RuV sequences. This analysis revealed an alternative Cp ^218^GIWG^221^ motif in several RuV sequences. Intriguingly, this sequence motif is also GIWG in RuhV (Fig. 8B). Taken together, our data provide biochemical and genetic evidence of Cp interactions with the E2 endodomain, and their conservation across other mammalian rubiviruses.

## DISCUSSION

We identified a potential late domain motif on the RuV E2 protein, ^278^PPAY^281^, and showed that alanine replacement of E2 Y281 severely inhibited RuV particle production. However, RuV was insensitive to the expression of dominant-negative VPS4A, and comparison of the RuV E2 sequence with those of the recently discovered rubiviruses showed that the E2 PPAY motif was not conserved. Revertants of the RuV E2 Y281A mutation demonstrated that a valine effectively substituted at this position, while the combined second-site mutations [E2 N277I + Cp D215A] also partially rescued. Thus, together, our data suggest that RuV (and by inference, other rubiviruses), is not dependent on the ESCRT machinery for budding. Instead, E2 Y281 was found to play an important role in Cp recruitment to the Golgi complex where virus budding occurs.

Our studies demonstrated that the structural proteins were comparably synthesized in both WT and E2 Y281A mutant-infected cells, indicating correct ER translocation and processing by signal peptidase. Formation of the E2-E1 heterodimer, glycosylation including acquisition of EndoH-resistance, and localization of E2 and E1 in the Golgi were similar between WT and mutant, in keeping with correct envelope protein folding and targeting to the Golgi by the E2 TM domain. These biochemical analyses and recognition of Cp, E2, and E1 by specific mAbs argue against a global misfolding of the E2 Y281A structural proteins. However, while the WT Cp colocalized with E2 and E1 in the perinuclear Golgi complex, in mutant-infected cells, the Cp was detected in a dispersed reticular pattern. In addition, cross-linking co-IP studies indicated that the E2 Y281A mutation disrupted Cp-envelope protein interaction. The effects of the E2 Y281A mutation were unexpected, as according to published literature, this region of E2 was believed to form a second TM domain at the E2 C-terminus (2, 11, 15), and was not previously implicated in Cp interactions or budding.

We performed bioinformatic analysis of the E2/E1 junction region. The results indicated that although the C-terminus of E2 encodes the E1SS and contains a signal peptidase cleavage site, it is not predicted to serve as a TM domain. It thus appears likely that the E1SS at the E2 C terminus reorients back into the cytoplasm after signal peptidase cleavage. This is reminiscent of the situation for alphaviruses (45, 46), members of the Togaviridae family in which RuV was formerly classified. The C-terminal region of alphavirus E2 serves as a signal sequence but is not maintained as a TM domain. A tyrosine-containing motif in the alphavirus E2 protein endodomain is critical for recruiting Cp to the budding site (47), and high-resolution structural analysis of alphavirus particles demonstrates the direct interaction of this motif with a hydrophobic pocket on the Cp (48, 49). While alphaviruses bud from the PM and their Cp do not contain a membrane anchor, they provide intriguing evidence for signal sequence reorientation and E2 endodomain-Cp interaction.

The recent discovery of RuhV in bats and RusV in mice and various other mammals (7–9, 50) expands the rubivirus genus and enables informative sequence comparisons. RuV forms a clade with RuhV, with RusV a close outgroup to this clade (7). The pseudorevertant of E2 Y281A has a V in the 281 position. The corresponding residues in RuhV and RusV are V and W, respectively. Thus the most efficiently rescued RuV revertant showed a relationship with the RuhV sequence. The position of the second-site revertant substitution E2 N277I corresponds to an I in RuhV and L, an isomer of I, in RusV. In addition, this revertant also required the Cp substitution Cp D215A, a position that is conserved as an acidic residue across these mammalian rubiviruses. While neither the pseudorevertant nor the second-site revertant completely restored the severely impaired Y281A growth, the rescue of RuV via substitutions present in the RuhV sequence provides experimental support for the close relationship of these two viruses.

Our results also support the importance of Cp-Golgi colocalization in RuV virus production, as the recovery of colocalization strongly correlated with rescue of growth. Prior studies demonstrated that the RuV Cp TM domain is needed for Cp-Golgi colocalization (14, 24). This could be mediated by interaction of the Cp membrane anchor with the E2 membrane anchor in the plane of the membrane, with the E2 TM domain providing the Golgi targeting information. Our data suggest that such an interaction would be insufficient for Cp targeting however, since the E2 Y281A mutation strongly blocks Cp transport to the Golgi. Instead, the rescue of growth and targeting by the second-site revertant containing both E2 N277I + Cp D215A suggests an important interaction between the E2 endodomain and the Cp cytoplasmic domain. Such an interaction is also supported by the cross-linking-co-IP of the WT Cp-E2, and its loss in Y281A and partial recovery in the revertants. The Cp TM domain could simply be acting to increase the local concentration of the Cp in the rough endoplasmic reticulum (RER) where the E2 protein is translocated. Alternatively, bivalent interactions between the E2 and Cp TM domains and the E2 endodomain and Cp cytoplasmic domain could together promote Cp transport to the Golgi and virus particle production.

The second-site revertant provided evidence for interaction between the E2 endodomain and the Cp cytoplasmic domain. Previous structural studies (25) proposed that a conserved arginine-rich E2 cytoplasmic region could interact with a detergent-containing pocket on Cp that is enriched with acidic and hydrophobic residues (indicated in Fig. 8B). The Cp mutation D215A was required in the second-site revertant, but inspection of the Cp structure indicates that this residue is distant from the putative binding pocket on Cp. Instead, Cp D215 is very close to the ^218^GVWG^221^ motif that was implicated in Cp interdimer interactions (25). Our data thus support an interaction of the reoriented endodomain of E2, involving E2 Y281, with the Cp cytoplasmic domain (Fig. 8C), perhaps promoted by changes in the Cp interdimer interface. An interaction of the E2 arginine-rich region with Cp could also play a role. Electron microscopy studies have shown that striking morphological changes occur during the transport of RuV particles through the secretory pathway, including rearrangement of the envelope proteins, condensation of the nucleocapsid core, and apparent changes in their interactions (17). These have been hypothesized to reflect a required RuV maturation process. We speculate that the E2-Cp interactions we discuss here may also play a role in these particle rearrangements.

## MATERIALS AND METHODS

### Cell lines and antibodies

BHK-21/WI-2, BHK-21/C-13, and Vero cells were cultured in Dulbecco’s modified Eagle’s medium (DMEM) containing 25 mM d-glucose, 4 mM l-glutamine, 100 U penicillin/mL, and 100 µg streptomycin/mL. Complete media included 10% fetal bovine serum (FBS) for Vero and 10% tryptose phosphate broth either 5% (BHK-21/WI-2) or 10% (BHK-21/C-13) FBS. Unless otherwise stated, BHK-21/WI-2 cells were used for most experiments; BHK-21/C-13 cells were only used for Fig. 2C and Fig. S3, to maximize electroporation efficiency (51). The T-REx-U-2 OS cell line expressing the tetracycline repressor (52) was cultured in modified McCoy’s 5A medium containing Hanks’ salts, 16 mM d-glucose, 10% FBS, 3.5 mM l-glutamine, 100 U penicillin/mL, 100 µg streptomycin/mL, and 60 µg hygromycin/mL. All cell lines were maintained at 37°C in 5% CO_2_. Tetracycline-inducible clonal T-REx-U-2 OS VPS4A-WT and VPS4A-EQ stable cell lines were generated as described previously (30, 53) and maintained in complete media plus 100 µg Zeocin/mL.

Commercial antibodies used were RuV polyclonal antibody (pAb) (MilliporeSigma catalog no. AB1060, Burlington, MA), RuV capsid-specific monoclonal antibody (mAb) (Genetex catalog no. GTX39194, Irvine, CA), RuV E2-specific mAb (ThermoFisher Scientific catalog no. MA5-18255, Waltham, MA), and GM130-specific mAb (Cell Signaling Technology catalog no. 12480, Danvers, MA). Mouse mAbs against RuV E2 (E2-1) and E1 (mAbs E1-20, E1-18) were a kind gift from Dr. Tom Hobman. The E1 mAb was used for co-IP analysis as the E2 mAbs were active in WB and IF but not IP.

### Viruses

The pBRM33 infectious clone plasmid (a gift from Dr. Tom Hobman) was used for the production of RuV and for generating RuV mutants for this study (54). The structural ORF was identical to GenBank ID OM816674.1. Briefly, the plasmids were linearized by *Hind III* and 750 ng of linearized plasmid was used as template in a standard 50 µL *in vitro* transcription reaction driven by SP6 polymerase (55). Unless otherwise stated, 15 µL of the unpurified transcription reaction was electroporated into BHK-21 cells using a Bio-Rad Gene Pulser and two consecutive 0.85 kV, 25 µF pulses in a 0.4 cm cuvette. The culture media were harvested 48 h post-electroporation and the virus titer was determined by infectious center assay (ICA).

### Generation of E2 P278A and Y281A mutants

Given the region’s high GC content (70%) and to facilitate cloning, the E2 C-terminal region of pBRM33 was cloned into pUC19 using the BamHI and SbfI sites to produce pUC19_BamHI_SbFI_M33. Alanine residues were substituted for E2 P278 or Y281 using PCR mutagenesis (56), and the mutagenized BamHI/SbfI fragments were ligated back into pBRM33. Two independent clones of each mutant were confirmed by Sanger sequencing (Genewiz, South Plainfield, NJ). Growth curves of the mutants were performed by electroporation of comparable amounts of *in vitro*-transcribed viral RNAs into BHK-21 cells. Progeny virus was titered by ICA.

Plasmids expressing the E2E1 glycoproteins were constructed as described recently (36). Briefly, the sequence from the RuV structural ORF (GenBank: OM816674.1) corresponding to aa 245–1,063 including a part of Cp with the E2SS (57) was codon optimized for mammalian expression and synthesized by Twist Bioscience (South San Francisco, CA). This was cloned into a mammalian expression vector and named pTWIST-RuV-245-E2E1. Using this plasmid, E2 Y281 was changed to A by standard PCR-based mutagenesis and termed pTWIST-RuV-245-E2Y281AE1. Plasmid sequences were confirmed by sequencing as above.

### Virus titration

The ICA and focus forming assay (FFA) were performed using a 96-well format. Briefly, 12,000 Vero cells were seeded per well and cultured overnight. Cells were then infected for 4 h with 100 µL/well of serial dilutions of RuV prepared in MEME containing 0.2% BSA, 2 mM l-glutamine, 10 mM HEPES pH 7.0, 100 U penicillin/mL, and 100 ug streptomycin/mL. Cells were washed and incubated for 48 h in 100 µL DMEM plus 5% FBS, and containing 20 mM NH_4_Cl (ICA) or 2% carboxymethylcellulose (FFA). Cells were fixed with 4% paraformaldehyde and stained with RuV pAb. The ICA was detected by staining with fluorescent-tagged secondary antibody and quantitated by fluorescence microscopy. The FFA was detected using a peroxidase-labeled secondary antibody and quantitated using an Immunospot S6 CTL analyzer.

### Effect of DN VPS4 expression

T-REx-U-2 OS cell lines inducibly expressing GFP-tagged VPS4A-WT or VPS4-EQ (30) were infected with RuV (MOI 15 IC/cell) for 4 h, followed by washing and additional incubation for 29 h. Cells were then washed and treated as indicated with 1 µg doxycycline/mL. Media were collected 1 and 13 h post-treatment and titered by ICA. VPS4-EQ-expressing cells were reproducibly observed to be healthy at 13 h post-induction. VSV infection was performed as reported previously (30). Briefly, VPS4A-WT or VPS4-EQ expression was induced for 1 h. Cells were then infected with VSV at an MOI of 10 PFU/cell for 1 h, washed, and media were collected 2.2 or 8 h post-treatment and titered by plaque assay on Vero cells.

### Virus particle production

BHK-21 cells were electroporated with the indicated viral RNAs and cultured in 10 cm plates for 42 or 48 h. About 8 mL of the culture media was harvested and pelleted through a cushion of 20% sucrose (wt/vol in 50 mM TRIS pH 7.4, 100 mM NaCl) in an SW41 rotor at 35,000 RPM for 3 h. The cell monolayer was lysed for 20 min on ice in 900 µL lysis buffer 1 (50 mM TRIS pH 7.4, 100 mM NaCl, 1 mM EDTA, 1% Triton X-100, 1 µg pepstatin/mL, 2 µg aprotinin/mL, and 1 mM PMSF) and then clarified by centrifugation. Lysates and virus pellets were analyzed by SDS-PAGE and western blotting with RuV pAb.

### Analysis of WT and mutant viral proteins in infected cells

Lysates of BHK-21 cells electroporated with WT, mutant, and no RNA were prepared as described above. To analyze glycoproteins using expression plasmids, Vero cells were transfected with E2E1 expression plasmids pTWIST-RuV-245-E2E1 (WT) or pTWIST-RuV-245-E2Y281AE1 or no plasmid using Lipofectamine 2000 (Invitrogen) according to the manufacturer’s instructions. To analyze glycosylation, aliquots of the lysates were denatured in the presence of DTT and SDS at 100°C for 10 min and then incubated at 37°C for 90 min with PNGase F or Endoglycosidase H according to the manufacturer’s protocol (NEB, Ipswich, MA).

To analyze E2-E1 dimer formation by co-IP, BHK-21 lysates were precleared by incubation with Protein-A agarose beads (ThermoFisher Scientific, Waltham, MA), then incubated with mAb E1-20 on ice for 1 h and retrieved with Protein A agarose beads by rotation for 90 min at 4°C. The beads were washed three times with lysis buffer 1 containing 0.1% Triton X-100, and resuspended in buffer containing 10 mM TRIS pH 6.8, 1 mM EDTA, 1 mM PMSF, and 2 µg aprotinin/mL and stored at −20°C. Before SDS-PAGE analysis, SDS and DTT were added to 0.5% and 40 mM, respectively, and samples were treated for 10 min at 100°C.

All samples were analyzed by SDS-PAGE and western blotting using RuV pAb or ThermoFisher E2 mAb as indicated in the legends.

### Selection and sequence characterization of E2 Y281A revertants

Vero cells from a confluent 10 cm dish were electroporated with 30 µL of *in vitro-*transcribed E2 Y281A RNA, distributed into ten 35 cm plates, and cultured for 10 days to select for revertants. Aliquots of 250 µL of the culture media were titered by ICA at 5 and 10 days post-electroporation. Samples in which virus growth was observed were expanded by culture on fresh Vero cells for another 10 days, when all samples had a titer of ~10^6^ IC/mL. Revertants were then isolated by serial dilution on 96-well plates and expanded on Vero cells.

RNA was extracted from approximately 10^6^ IC of each potential revertant using the RNeasy mini kit (Qiagen, Hilden, Germany), eluted in 30 µL H_2_0, and reverse transcribed using the ProtoScript II First Strand cDNA Synthesis Kit (NEB, Ipswich, MA), all according to the manufacturer’s protocols. The structural protein ORFs were amplified by PCR of overlapping fragments using appropriate primer pairs and Q5 high-fidelity polymerase (NEB, Ipswich, MA) with high GC enhancer supplement. Samples were sequenced by Sanger sequencing (Genewiz, South Plainfield, NJ) and the sequences were analyzed using BioEdit (58). To analyze the effects of individual or pairs of mutations, PCR fragments carrying the mutation/s were ligated into the pBRM33 Y281A infectious clone and confirmed by sequencing.

### Confocal microscopy analysis

Vero cells were seeded on coverslips and transfected for 6 h with WT or mutant RNAs using Lipofectamine 2000. Cells were then washed and cultured in fresh media for another 36 h. Cells were fixed with 4% paraformaldehyde, permeabilized, and stained for 50 min with the following mAbs: (Fig. 3A) mAb E1-18 and E2-1 (1:50); (Fig. 3B and 5B) E2-1 (1:50) and Cp mAb (1:750); (Fig. 4) Cp mAb (1:750) and GM130 mAb (1:3000); (Fig. S2) mAb E1-20 (1:100) and Cp mAb (1:750). Secondary staining used appropriate isotype-specific antibodies conjugated with Alexa Fluor dye and diluted 1:350, and Hoeschst dye (ThermoFisher Scientific, Waltham, MA) diluted 1:10,000. Images were acquired using a 100× oil objective on the Nikon CSU-W1 Spinning Disk confocal microscope in the Einstein Analytical Imaging Facility. Z-stacks were acquired at 0.2 µm step size for the entire depth of the cell. A total of 15–20 randomly chosen cells were imaged for each condition. The Pearson’s correlation coefficients (*r*) were calculated using Volocity 7.0 (PerkinElmer) and figures were prepared using the ImageJ QuickFigures plugin (NIH) and Inkscape.

### Co-immunoprecipitation of capsid by E2E1 dimer

BHK-21 cells were electroporated with WT or mutant RNAs and cultured in 15 cm plates for 42 h. Cell monolayers were then washed with phosphate-buffered saline (PBS), followed by cross-linking at room temperature for 10 min in the dark using 1 mM dithiobis succinimidyl propionate (DSP, ThermoFisher catalog no. 22585) prepared in PBS. The cells were then washed once with quenching buffer (125 mM glycine prepared in PBS) and incubated in quenching buffer for additional 10 min. The cells were then washed with PBS, scraped, pooled, and pelleted by centrifugation. The cell pellets were lysed in lysis buffer 2 (10 mM sodium phosphate pH 7.4, 50 mM NaCl, 1% Triton X-100, 1 µg pepstatin/mL, 2 µg aprotinin/mL, and 1 mM PMSF) on ice for 20 min and clarified by centrifugation. Lysates were then processed as described for the E2-E1 co-Ip with the following modifications: Equal volumes of precleared lysates were incubated with mAb E1-20 or a control mAb. The mAbs were captured by Agarose A beads and washed three times with lysis buffer 2 with 0.1% Triton X-100 and resuspended in buffer containing 10 mM TRIS pH 7.4, 1 mM EDTA, 1 mM PMSF, and 2 µg aprotinin/mL. For DSP cleavage and sample denaturation, SDS and DTT were added to 0.5% and 40 mM, respectively, and samples were treated for 5 min at 100°C before gel analysis.

### Prediction of transmembrane domains and signal sequences in RuV structural proteins

The full-length RuV structural polyprotein (1,063 residues) was scanned for the presence of TM helices using the ΔG for TM helix insertion server (39). A negative ΔG value for a region suggests that it is recognized by the Sec61 translocon and integrated into the ER membrane. As an independent approach, we also used the TOPCONS (41) and Phyre2 (42) servers to predict the TM region. The TOPCONS algorithm combines topology predictions and evaluates their level of agreement, while Phyre2, a structure-based homology modeling server, predicts by matching the query sequence to a library of known protein structures.

SS were predicted by using the SignalP-6.0 server, which predicts standard secretory signal peptides transported by the Sec translocon and cleaved by signal peptidase (43). We identified high probability SS- and cleavage site-carrying sequences by examining several 70 aa windows that include the published Cp/E2 and E2/E1 cleavage sites.

### Rubivirus sequence comparisons

The structural protein sequences of these rubiviruses (accession numbers listed) were used for sequence analysis: RuV (P08563, 1,063 aa), RuhV (QKO01647.1, 1,098 aa), and RusV (QKO01649.2, 1,143 aa). Given that the structural protein ORFs of the three rubiviruses are of different lengths and the exact N- and C-termini of Cp, E2, and E1 are not yet experimentally determined for the recently discovered rubiviruses (7, 8), the termini were inferred by first aligning the full- length structural ORFs of RusV and RuhV to that of RuV. For the final analysis shown in Fig. 8A and B, the structural proteins were individually aligned. This strategy was helpful in avoiding compensatory gaps in the reference RuV sequence.

## SUPPLEMENTAL MATERIAL

Fig. S1 mbio.01965-24-s0001.tiff Expression of VPS4A-WT and VPS4A-EQ.

Fig. S2 mbio.01965-24-s0002.tiff Localization of E1 and Cp proteins.

Fig. S3 mbio.01965-24-s0003.tiff Structural proteins ratios of revertant.

Legends mbio.01965-24-s0004.docx Supplemental figure legends.

ASM does not own the copyrights to Supplemental Material that may be linked to, or accessed through, an article. The authors have granted ASM a non-exclusive, world-wide license to publish the Supplemental Material files. Please contact the corresponding author directly for reuse.
