# Supplementary figures and images for: Rubella virus assembly requirements and evolutionary relationships with novel rubiviruses
Source: mBio. 2024 Aug 29;15(10):e01965-24. doi: 10.1128/mbio.01965-24 (PMC11481484; doi:10.1128/mbio.01965-24)

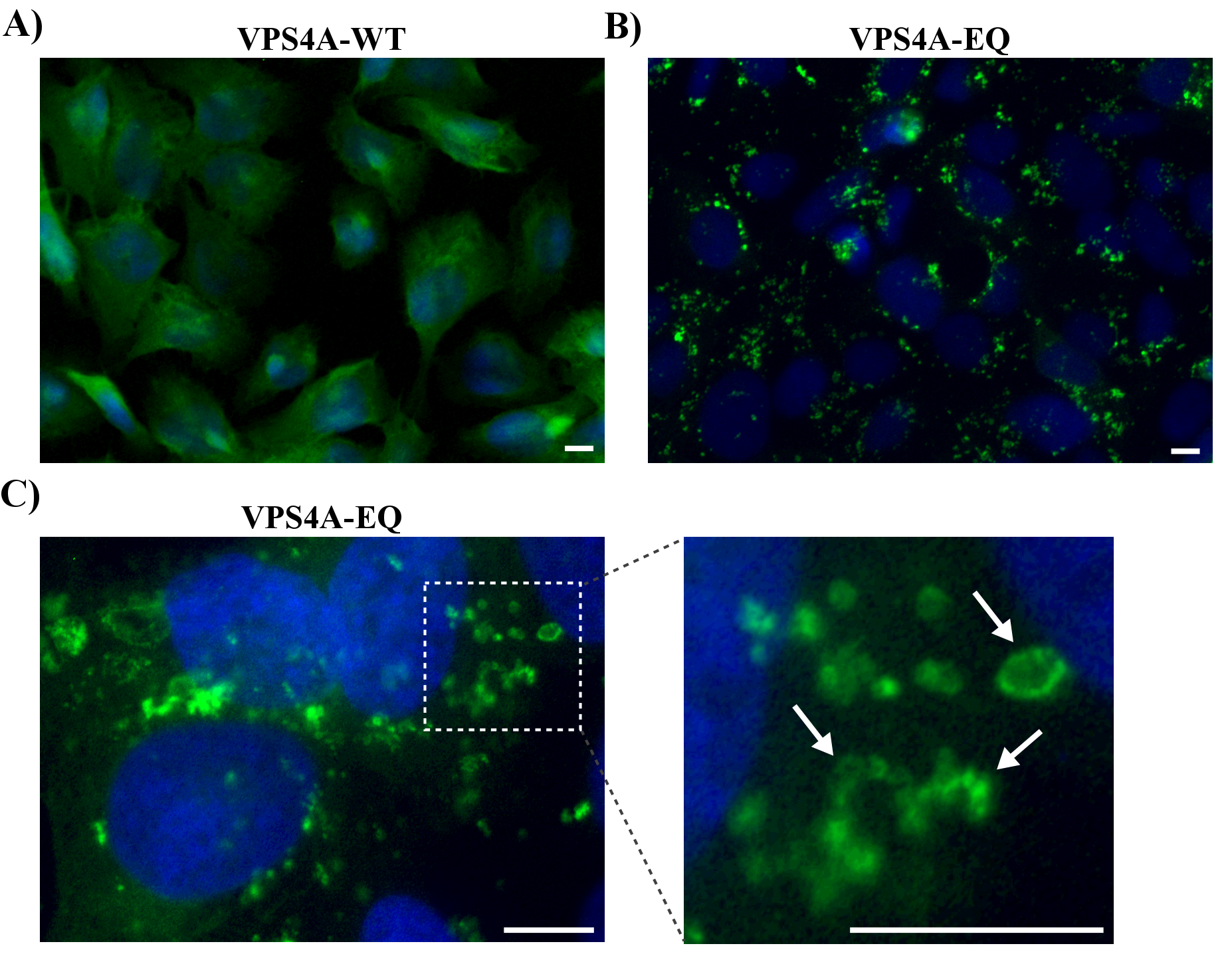

Supplement: Fig. S1 — Expression of VPS4A-WT and VPS4A-EQ. [file mbio.01965-24-s0001.tiff]

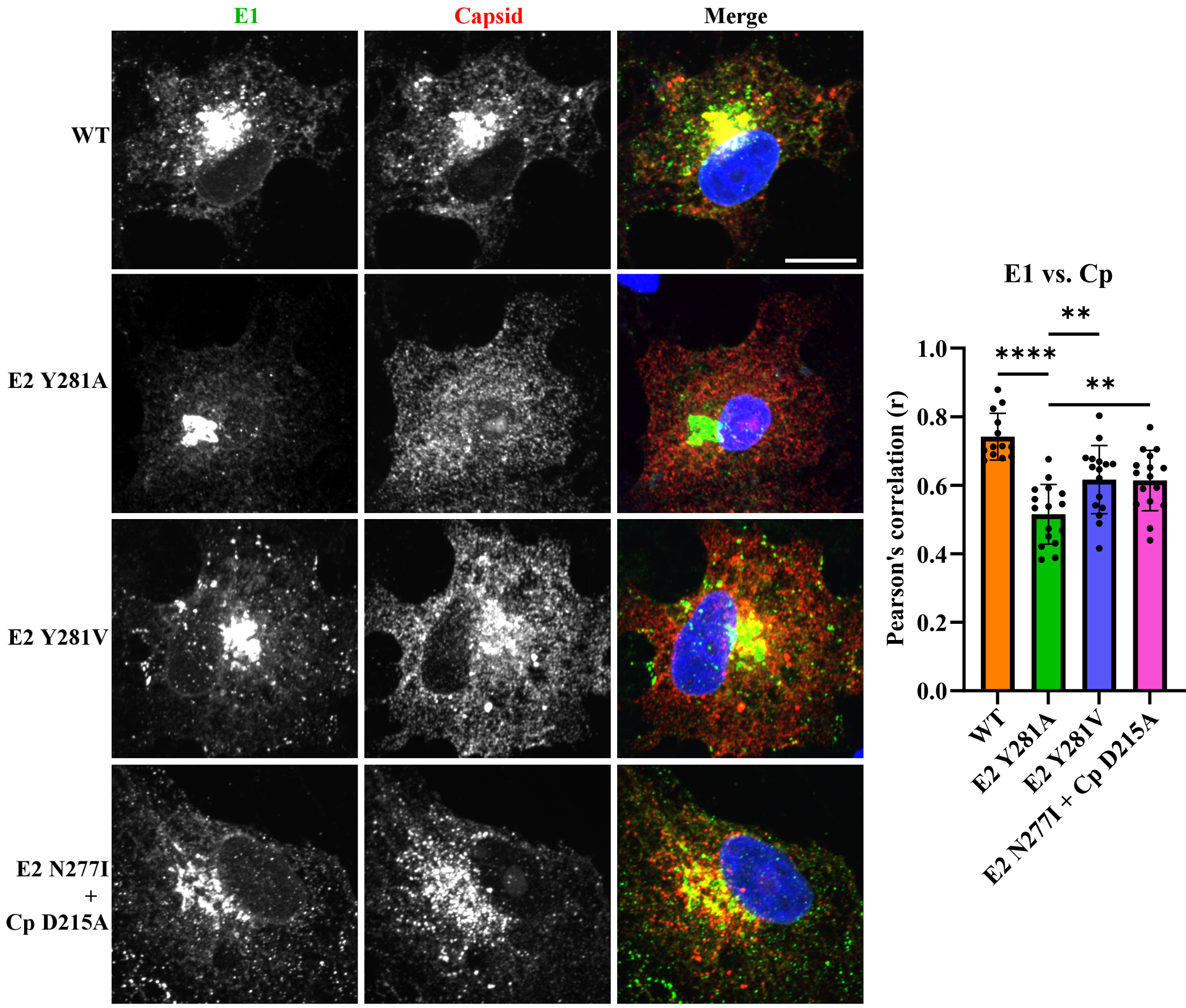

Supplement: Fig. S2 — Localization of E1 and Cp proteins. [file mbio.01965-24-s0002.tiff]

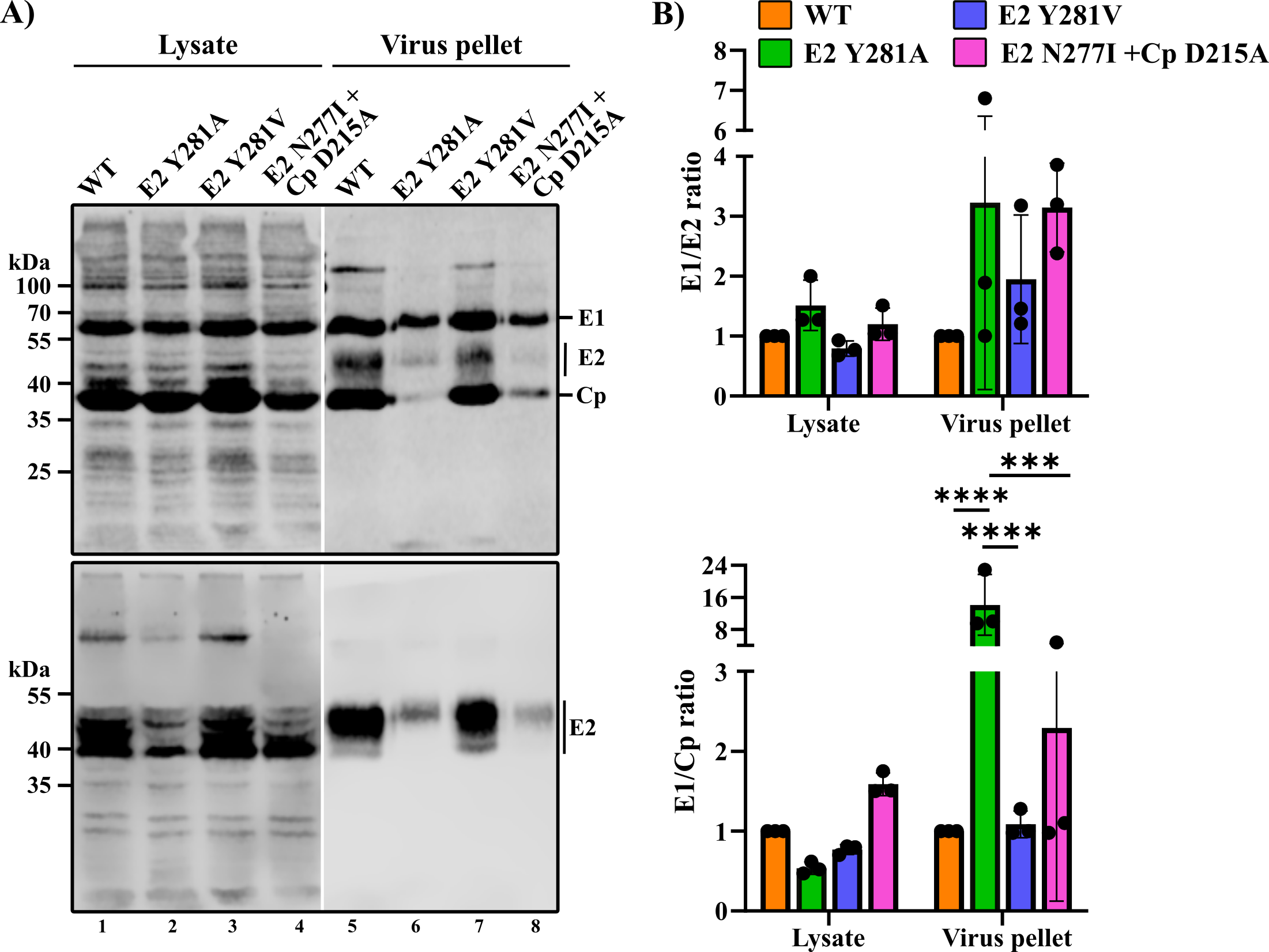

Supplement: Fig. S3 — Structural proteins ratios of revertant. [file mbio.01965-24-s0003.tiff]
