# Supplementary material for: Rubella virus assembly requirements and evolutionary relationships with novel rubiviruses
Source: mBio. 2024 Aug 29;15(10):e01965-24. doi: 10.1128/mbio.01965-24 (PMC11481484; doi:10.1128/mbio.01965-24)
Supplement: Legends — Supplemental figure legends. [file mbio.01965-24-s0004.docx]

**Supplementary Figure Legends (Figure S1-S3)**

**Figure S1. Expression of VPS4A-WT and VPS4A-EQ.** Clonal T-REx-U-2-OS cell lines were cultured in 1 µg doxycycline/mL for 16 h to induce expression of GFP-tagged VPS4A-WT or VPS4A-EQ. Samples were imaged with 20x (A and B) or 63x (C) objectives using a Zeiss Axiovert 200M microscope. Consistent with previous observations (1) VPS4A-WT remained cytoplasmic (A) while expression of VPS4A-EQ produced labeled vesicular structures (B and C). The region indicated by the white dotted box in panel C was magnified (~3x) to show the GFP-labelled vesicular compartment (white arrows). Images are representative of three independent experiments. Scale bar = 10 μm.

**Figure S2. Localization of E1 and Cp proteins.** Vero cells were transfected with RNA from RuV WT or the indicated mutant or revertant. At 42 h post transfection cells were fixed, permeabilized and stained with mAbs to E1 and Cp and with Hoechst to visualize the nuclei. Samples were imaged by confocal microscopy. Images are a representative example from two independent experiments. The bar graph in the right panel shows the Pearson’s correlation coefficient (r) determined for 10-15 cells/sample from one experiment, showing the individual data points and the mean and standard deviation. Statistical analyses were carried out by one-way ANOVA with Dunnett's multiple comparisons test. ****, P< 0.0001; **, P< 0.01. Scale bar = 10 μm.

**Figure S3. Structural proteins ratios of revertant.** (A) BHK-21 cells were electroporated with WT or indicated mutant RNAs and cultured for 48 h. Cell lysates were prepared and the virus from the 48 h culture media was pelleted through a sucrose cushion. All samples were analyzed by western blotting with RuV pAb (upper panel) or E2 mAb (lower panel). The white space between lanes 4 and 5 indicates where two separate blot images from the same experiment have been combined. (B) Quantification of E1/E2 (upper panel) or E1/Cp (lower panel) ratio from experiments performed as in panel A (n=3). Statistical analyses were carried out by two-way ANOVA with Dunnett's multiple comparisons test against E2 Y281A. ****, P< 0.0001; ***, P< 0.001.

**References**

1. Lin Y, Kimpler LA, Naismith TV, Lauer JM, Hanson PI. 2005. Interaction of the mammalian endosomal sorting complex required for transport (ESCRT) III protein hSnf7-1 with itself, membranes, and the AAA+ ATPase SKD1. J Biol Chem 280:12799–12809.
